# Anti-distraction learning: focused attention, task engagement, and flow under cognitive interference

**DOI:** 10.3389/fpsyg.2026.1795811

**Published:** 2026-04-01

**Authors:** Shengying Yang, Sinian Chen, Jing Gui, Pinxi Liu

**Affiliations:** 1 School of Computer Science and Technology, Zhejiang University of Science and Technology, Hangzhou, Zhejiang, China; 2 Belarusian State University, Minsk, Belarus; 3 College of Arts, Media and Technology, Chiang Mai University, Chiang Mai, Thailand; 4 School of Humanities and Arts, Guangxi Vocational University of Agriculture, Nanning, China

**Keywords:** cognitive interference, environmental noise, flow experience, focused attention, immersion, task engagement

## Abstract

Flow experience is often described as a psychological state that is easier to sustain when external disruption is limited. In many contemporary learning and work environments, however, individuals frequently perform tasks amid notifications, background noise, and task switching. Although existing studies have documented the disruptive effects of interference on task performance, comparatively less attention has been paid to how immersive experience may still emerge when interruptions are persistent rather than occasional. The present study therefore examines how cognitive interference and environmental noise relate to flow experience while distinguishing the roles of focused attention and task engagement as regulatory processes within this relationship. Questionnaire data were collected from individuals engaged in everyday learning activities (*N* = 647). Cognitive interference and environmental noise were modeled as contextual conditions, focused attention and task engagement as mediating mechanisms, and flow experience as the outcome variable. Hierarchical regression analysis, bootstrap mediation tests, and alternative model specifications were implemented within a scripted analytical environment to evaluate the proposed relationships. The results indicate that both cognitive interference and environmental noise remain significantly associated with flow experience even after attentional and behavioral regulatory mechanisms are included in the model. Focused attention shows the strongest association with flow and mediates the relationship between cognitive interference and immersive experience, suggesting that attentional stabilization plays a central role in sustaining immersion under disruptive conditions. Task engagement contributes to continued task activity but shows a smaller and negative indirect pathway to flow, indicating that behavioral persistence does not necessarily translate into experiential immersion. In addition, the sequential mediation pathway linking interference, focused attention, task engagement, and flow experience is not supported. Taken together, these findings suggest that immersive experience can persist even under continuing disruption. Rather than depending solely on distraction-free environments, flow in contemporary learning contexts may emerge through repeated stabilization of attention during ongoing interruptions. Attentional regulation preserves experiential continuity, while task engagement sustains behavioral continuity, together allowing immersion to remain achievable in interference-rich environments.

## 1 Introduction

### 1.1 Immersive experience under conditions of persistent interference

Background interference has become a routine element of task activity in many learning and work environments. Many classrooms now operate with continuous ambient sound, and open-plan offices expose workers to overlapping speech, device alerts, and intermittent notifications. Under these conditions, uninterrupted task periods are short and frequently disrupted. Noise in such settings functions not only as an environmental backdrop but as a condition that shapes how attention is allocated and cognitive effort is experienced. Research in education and occupational settings has documented similar patterns. Klatte et al. (2013) reported elevated noise levels in schools and linked them to reduced learning efficiency. Beaman (2005) showed that relatively low-intensity background noise can interfere with sustained attention and lower the likelihood of maintaining stable engagement. Open-plan office layouts further intensify these attentional demands by increasing exposure to background speech and acoustic disturbance. Bergefurt et al. (2024) connected such exposure to distraction, psychological fatigue, and increased coping effort. Comparable associations have been reported across different sectors, although the magnitude and mechanisms vary by context. Experimental studies also indicate that noise interrupts complex cognitive processing. Furnham and Strbac (2002) found that background sound increases off-task thought and goal neglect during ongoing work. Zhou et al. (2022) showed that broadband noise alters perceived workload and disrupts the rhythm of decision-making and task execution. Interference therefore influences task performance and weakens the psychological basis required for sustained immersion. When disruption becomes routine, immersion is more difficult to maintain and depends on active attentional regulation. This places interference at the center of practical challenges in classrooms and offices and positions it as a critical test case for theories of attention and immersive experience.

### 1.2 Connections among flow, attention, and task engagement in existing research

Early theoretical work on immersive experience emphasized deep concentration rather than interference. Initial studies focused on how sustained engagement emerges when situational challenge aligns with individual capacity. As digital learning environments became more common, researchers increasingly began to examine how immersion can be supported through designed learning conditions. Bian et al. (2022) showed that reducing external distraction strengthens the association between flow experience and learning performance, suggesting that environmental conditions can shape the likelihood of immersive experience during learning activities. This shift gradually redirected flow research from describing subjective phenomenological states toward examining the contextual and psychological processes that support immersion. In parallel, research on attention control provided a cognitive explanation for how immersion may be maintained during task activity. Neurocognitive findings reported by Hopf et al. (2002) indicated that attentional systems enhance goal-relevant information while suppressing competing input, thereby stabilizing perceptual processing. This evidence reframed attention as an active selective system rather than a passive mental resource. Sörqvist et al. (2016) further suggested that when cognitive load is appropriately engaged, attentional mechanisms can help shield task processing from external distraction, making concentration an actively regulated state. Studies conducted in everyday learning contexts later emphasized the role of task engagement in linking attention and immersive experience. Parker and Heywood (2013) observed that continuous participation and reflection during task activity support deeper meaning construction, indicating that engagement is better understood as a sustained pattern of behavioral and cognitive involvement rather than a brief moment of focus. As mobile devices and frequent information prompts became routine features of contemporary learning environments, maintaining engagement under conditions of persistent interruption attracted increasing scholarly attention. Aagaard (2021) reported that students adopt self-monitoring, environmental adjustment, and behavioral strategies to manage distraction, suggesting that task engagement can function as a learnable form of self-regulation during task activity. Taken together, these research strands suggest that immersive experience does not depend solely on the absence of distraction but may also be supported through attentional regulation and sustained engagement during task performance. This perspective highlights the potential roles of attention and task engagement in sustaining flow even when learning occurs in environments characterized by ongoing disruption.

### 1.3 Pathways linking cognitive interference to flow experience

Although interference is typically associated with disrupted performance, everyday task environments rarely eliminate distraction entirely. Under such conditions, individuals may rely on attentional regulation to stabilize task processing despite ongoing interference. In learning and work environments where interference is routine, cognitive neuroscience has devoted sustained attention to how cognitive systems withstand distraction. Initial studies concentrated on how interference alters basic cognitive operations. Dolcos and McCarthy (2006) reported that emotional distraction engages neural systems associated with reduced maintenance of working memory. Anticevic et al. (2010) also found that resisting negative distraction draws on additional neural resources, suggesting that interference introduces internal regulatory demands alongside external stimulation. Reviewing auditory distraction, Campbell (2005) argued that interference can continuously draw on attentional capacity, making sustained target processing more vulnerable to disruption. Later work shifted toward attentional control as the mechanism linking interference and task performance. Parmentier and Hebrero (2013) showed that brief deviant sounds can trigger involuntary attentional shifts, and that re-orienting attention to the target task requires additional cognitive control. This suggests that interference does not only interrupt task processing but also activates regulatory mechanisms that allow individuals to redirect attention back to goal-relevant activity. Recent studies have further examined interference in applied contexts. Misra et al. (2023) reported that cognitive distraction during driving can be detected and used to predict behavioral deviation risk, demonstrating that interference effects extend beyond laboratory tasks and influence complex task execution over time. Even with these advances, the literature has largely emphasized how interference disrupts task execution. Comparatively fewer studies have examined how individuals sustain immersive experience under disruptive conditions. Flow research provides a well-established account of immersion, yet interference research has tended to operationalize outcomes primarily in terms of errors, reaction time, or workload. As a result, the two research traditions remain only loosely connected at the empirical level. When interference is persistent rather than occasional, individuals may repeatedly mobilize attentional resources to restore task stability, creating conditions under which immersion may still occur intermittently despite ongoing distraction. This observation raises an important question: when interference cannot be avoided, how is immersive experience maintained through attentional regulation and task engagement? Addressing this question places interference research, attention control theory, and flow theory within a single analytical framework, allowing the interaction among disruption, attentional regulation, and behavioral engagement to be examined simultaneously. The present study therefore investigates how cognitive interference relates to flow experience through attentional maintenance and task engagement and evaluates these pathways in everyday learning settings.

In interference-rich environments, greater perceived interference may not simply indicate cognitive breakdown; it may also reflect contexts in which individuals are required to mobilize regulatory resources more actively. Under such conditions, flow may emerge not because interference is beneficial in itself, but because immersive experience becomes dependent on repeated attentional recovery during ongoing task performance. Despite a growing body of research examining flow experience and distraction in learning environments, several important gaps remain. Much of the existing literature has treated environmental noise and cognitive interference primarily as factors that disrupt performance, focusing on outcomes such as errors, workload, or reduced efficiency, while comparatively less attention has been paid to how immersive experience may still emerge under persistent interference in everyday learning contexts. In addition, although attention control and task engagement have both been discussed as mechanisms supporting sustained task performance, these processes are often examined separately rather than integrated within a unified analytical framework, leaving the ways in which attentional regulation and behavioral engagement jointly shape flow experience under disruptive conditions insufficiently understood. Furthermore, empirical studies that connect interference research with flow theory in naturalistic learning environments remain relatively limited. At the same time, contemporary learning environments rarely eliminate distraction entirely, as learners frequently encounter background noise, digital notifications, and competing cognitive demands during task activity. In such contexts, individuals may repeatedly mobilize attentional resources to stabilize task processing and maintain engagement despite ongoing interference, suggesting that immersive experience may not depend solely on distraction-free environments but may instead emerge when attentional regulation temporarily restores cognitive stability during task execution. To address these gaps, the present study examines how cognitive interference relates to flow experience through the regulatory roles of focused attention and task engagement and evaluates these mechanisms using empirical data collected from everyday learning situations. Based on this perspective, the following hypotheses are proposed.

## 2 Literature review

### 2.1 Cognitive interference and environmental noise as conditions of disrupted performance

Early work on interference and task performance often framed the issue in terms of cognitive load. Ayres (2006) reported that lowering intrinsic load in learning tasks is associated with improved performance, suggesting that interference reflects additional cognitive occupation beyond task difficulty alone. Later multimedia-learning studies showed that proactive and retroactive interference can alter the order in which information is processed, as new content and prior knowledge compete for processing priority, potentially influencing memory formation and comprehension processes. Mayer et al. (2007) further demonstrated that identical learning content presented in different formats can generate distinct interference patterns and lead to different learning outcomes. In this line of research, interference was therefore interpreted less as purely external disturbance and more as a consequence of how internal cognitive processing is structured. Work in naturalistic learning and communication settings has similarly identified linguistic complexity as a source of interference. Brown et al. (2019) reported that complex scientific language requires substantial cognitive resources before conceptual understanding is achieved, which can place additional demands on processing capacity. Interference may therefore arise even in the absence of environmental noise when information itself requires extensive cognitive decoding. Research on environmental noise has also received sustained attention in experimental psychology and perceptual learning. Gold et al. (2004) showed that external noise can change how perceptual learning unfolds over time, leading individuals to adopt different adaptive strategies under noisy conditions. Applied studies further demonstrate that background speech, including semantically irrelevant content, forces attention to shift between linguistic input and task-related processing, increasing distraction-related processing costs (Marsh & Jones, 2010). More recent research has expanded the concept of noise beyond purely acoustic disturbance. Digital notifications and mobile alerts, for example, can interrupt attentional continuity and impose measurable cognitive costs during ongoing tasks (Stothart et al., 2015). Similarly, studies of contemporary work environments indicate that environmental sound conditions in open or activity-based workplaces shape distraction levels and cognitive performance (Jahncke and Hallman, 2020). Interference has also been examined in clinical and fatigue-related cognitive research, where Johnson et al. (1998) found that distraction is associated with increased memory difficulty among cognitively fatigued individuals, suggesting that processing sequences may become less stable under strain. Because disruption sources are diverse, the concept of environmental noise in learning research has gradually broadened. Some scholars conceptualize noise as any external stimulus that competes for attentional resources during task execution (Sörqvist and Rönnberg, 2014), while others emphasize that noise operates as a contextual condition that increases distraction and requires additional coping or cognitive regulation from individuals (Belojevic et al., 2003). Recent research in workplace environments further highlights how acoustic conditions influence distraction and coping processes in open-plan settings (Bergefurt et al., 2024). In the present study, environmental noise is therefore defined as background auditory or sensory disturbance—such as conversations, device notifications, or ambient sound—that competes for attentional resources and may interrupt ongoing cognitive processing during learning activities. Overall, existing findings suggest that cognitive interference and environmental noise function as contextual conditions that influence how attention is allocated, how processing sequences unfold, and how cognitive resources are distributed during task performance. Their effects extend beyond accuracy and reaction time and shape whether individuals are able to maintain stable processing and remain engaged with ongoing tasks. This perspective motivates examining how interference, through attentional regulation and task engagement, relates to the emergence of sustained immersive experience.

### 2.2 Focused attention as a regulatory process under interference

As evidence accumulated on how disruption relates to task performance, researchers began to examine how goal-directed processing is maintained under interference. This shift placed the attentional system at the center of explanations for how individuals regulate distraction during task activity. Early memory research treated interference as modifiable through internal regulation rather than as an unavoidable loss of processing capacity. Bower et al. (1994) showed that reorganizing retrieval cues can reduce the impact of retroactive interference on ongoing tasks, suggesting that attentional control operates not only at the level of perceptual selection but also in the organization of memory processes. Neurocognitive research further mapped mechanisms supporting attentional maintenance. Han et al. (1998) found that specific brain regions contribute to reducing proactive interference, helping stabilize information configuration during learning. This line of evidence framed attention as a sustained regulatory condition supporting complex learning structures rather than merely a brief moment of perceptual focus. Work on short-term maintenance added further detail to these processes. Avons and Sestieri (2005) reported that visual short-term memory can be preserved under conditions of dynamic visual noise, implying that attentional mechanisms are sometimes able to separate external noise input from internal representations and thereby limit direct disruption. Studies conducted in real behavioral contexts have similarly emphasized the active nature of attentional regulation. Dhooge and Hartsuiker (2011) demonstrated that during language production speakers rely on internal control processes to resist distraction and maintain continuity in speech planning, supporting the view that attention functions as an active regulatory system rather than as a resource that is simply depleted by interference. Research on auditory distraction has also highlighted the relationship between working memory and attentional capacity. Sörqvist (2010) argued that individuals with higher working memory capacity are better able to sustain attentional stability, which can reduce performance fluctuation under auditory distraction. This line of work suggests that attentional control plays an important role in explaining individual differences in how interference is managed. Language-learning research reports similar patterns. Tocalli-Beller and Swain (2005) observed that when learners encounter cognitive conflict during language reconstruction tasks, redirecting attention can stimulate more elaborative processing. In this sense, interference does not necessarily lead to cognitive breakdown; under conditions of effective attentional regulation, it may also coincide with deeper processing activity. Taken together, these findings suggest that focused attention is better understood as a dynamic regulatory process rather than simply the absence of distraction. Attention guides how cognitive resources are allocated, constrains the influence of interfering input, and helps maintain continuity of processing under unstable conditions. This perspective provides a basis for examining how attentional regulation may shape the relationship between interference and immersive experience.

### 2.3 Task engagement as a behavioral and cognitive response to disruptive contexts

After attentional regulation became a dominant explanation for how individuals manage interference, researchers increasingly turned their attention to how goal-directed activity continues under conditions of persistent disruption. Within this line of work, task engagement was gradually conceptualized as a sustained pattern of behavioral and cognitive participation rather than a brief moment of psychological focus. Scholarly attention therefore shifted from asking whether distraction occurs to examining how individuals resume and maintain task activity after interruptions. In learning contexts, task engagement has often been described through self-regulatory practices. Aagaard (2021) reported that students in digital learning environments respond to interference by organizing study spaces, managing device notifications, and monitoring attentional states in order to maintain task continuity. These behaviors frame task engagement as a set of concrete maintenance practices rather than merely a subjective feeling of concentration. Evidence from motor-skill learning research similarly indicates that interference can shape engagement patterns directly. Boutin and Blandin (2010) observed that contextual interference requires learners to adjust motor planning and execution strategies, which can support the development of stable skill structures. In this account, engagement involves more than resisting disruption; it also reflects adaptive adjustments in task performance that contribute to skill development. Engagement patterns also vary across individuals and situations. Jing et al. (2012) found that under conditions of background music and noise, gender groups differed in task persistence and recovery speed following distraction, suggesting that engagement reflects both personal characteristics and environmental context. In research on attention-related learning difficulties, the behavioral significance of engagement is particularly visible. Kercood and Grskovic (2010) showed that reducing audiovisual distractions and guiding students to establish sustained participation strategies can improve mathematical task performance among learners with attention deficits, positioning task engagement as a behavioral maintenance mechanism that can be supported through instructional intervention. Recent work has also examined how individuals interpret the experience of distraction itself. Senn and Radomsky (2015) reported that beliefs about the meaning of distraction influence whether individuals return to tasks, indicating that engagement includes both behavioral maintenance and internal appraisal of interference. Similar patterns appear in complex professional activities. Williams and Drew (2017) found that brief interruptions can disturb search-strategy organization in medical image diagnosis, while rapid restoration of task rhythm contributes to reliable execution. Taken together, these findings suggest that in everyday environments, where distraction is difficult to eliminate entirely, the critical issue is not whether interruptions occur but how individuals reorganize behavior and resume task activity after disruption. In this sense, attentional regulation stabilizes processing in the moment, whereas task engagement supports the longer-term behavioral continuity of task performance.

### 2.4 Flow experience as an outcome of attention and engagement dynamics

With attentional regulation and task engagement increasingly recognized as core processes for maintaining performance under conditions of interference, recent work has turned to the experiential dimension of sustained activity—namely, whether individuals can enter and maintain immersion in complex environments. Flow is often regarded as a psychological marker of learning and work quality, capturing sustained concentration, intrinsic motivation, and the altered perception of time during task activity. Digital learning research has examined the relationship between flow and interference in empirical settings. Bian et al. (2022) reported that when external distractions are reduced, the association between flow experience and learning performance becomes stronger. This finding suggests that immersive experience is not determined solely by task design but is also shaped by the level of environmental disturbance and the stability of attentional focus during task execution. At the same time, a substantial body of research has shown that interference can disrupt the cognitive conditions required for stable task experience. In auditory cognition research, Campbell (2005) demonstrated that persistent auditory distraction draws on attentional capacity and makes sustained internal processing rhythms harder to maintain. Similarly, Zhou et al. (2022) found that broadband noise alters perceived cognitive workload and reduces the stability of decision rhythm and behavioral organization. These findings indicate that the emergence of flow depends not only on the balance between challenge and skill but also on whether attentional resources remain sufficiently stable under disruptive conditions. Evidence from complex real-world tasks further illustrates this issue. Misra et al. (2023) reported that cognitive distraction during driving can be detected and used to predict behavioral deviation risk, suggesting that interference can destabilize task execution structures over time. When task execution is repeatedly interrupted, immersive task experience becomes increasingly difficult to sustain. From this perspective, interference appears to undermine the stability of task engagement and the experiential conditions associated with flow. However, existing research also indicates that individuals do not simply disengage from tasks when interruptions occur. Studies on task engagement suggest that individuals often maintain task involvement by regulating attention and reorganizing behavior in response to disruptive conditions. Parker and Heywood (2013) observed that sustained participation and reflection during task activity support deeper meaning construction and coherence in learning experiences. Similarly, Aagaard (2021) reported that students in digital learning environments preserve task continuity through self-monitoring and environmental management strategies, suggesting that behavioral engagement can help maintain task activity even when distractions are present. These findings imply that individuals frequently respond to interference not by abandoning tasks but by mobilizing regulatory processes that stabilize attention and sustain task participation. From this perspective, flow may be better understood not only as a psychological state that emerges in distraction-free environments but also as a dynamic experiential condition supported by attentional regulation and sustained task engagement. Attentional regulation helps stabilize cognitive processing in the presence of competing stimuli, whereas task engagement maintains the behavioral continuity of task execution over time. In learning environments where interruptions cannot be entirely avoided, the coordination between attentional control and behavioral engagement may therefore shape whether immersive experience can still emerge during task activity. In such contexts, interference may not simply eliminate immersion but instead create conditions in which individuals must actively regulate attention and sustain task involvement to maintain task experience.

Building on this perspective, the present study examines how cognitive interference relates to flow experience through the regulatory roles of focused attention and task engagement in everyday learning settings. Specifically, it evaluates whether attentional regulation and behavioral engagement serve as pathways linking interference-related conditions to immersive task experience. In doing so, the study does not assume that interference is inherently beneficial. Rather, it argues that in environments where disruption is routine, higher perceived interference may coincide with stronger regulatory mobilization, thereby making immersive experience more likely to emerge episodically during task activity. On this basis, the following hypotheses are proposed:

*H1*: Cognitive interference is positively associated with flow experience.

*H2*: Environmental noise is positively associated with flow experience.

*H3*: Focused attention mediates the relationship between cognitive interference and flow experience.

*H4*: Task engagement mediates the relationship between cognitive interference and flow experience.

*H5*: Focused attention and task engagement form a sequential pathway linking cognitive interference to flow experience.

## 3 Materials and methods

### 3.1 Research design

This study adopts a quantitative cross-sectional design to examine how individuals maintain task experience under conditions of cognitive disruption. Rather than treating interference as a minor environmental factor, the study conceptualizes perceived disruption as a key contextual condition influencing attentional regulation, task engagement, and flow experience during task performance. The analytical framework focuses on cognitive and behavioral processes associated with specific task episodes rather than on stable personal traits. Within this framework, two contextual variables were modeled as exogenous constructs: cognitive interference (X1) and environmental noise (X2). Cognitive interference refers to perceived distraction, interruptions, and competing cognitive demands that occur during task execution. Environmental noise refers to background auditory or sensory disturbances in the surrounding environment that may disrupt concentration. Two regulatory processes were modeled as mediators. Focused attention (M1) represents the degree to which individuals maintain attentional stability and resist distraction during task activity. Task engagement (M2) reflects behavioral persistence, effort investment, and the tendency to resume task activity following interruptions. Flow experience (Y) was modeled as the outcome variable and captures immersion, altered time perception, and intrinsic involvement in the task. The questionnaire items used to measure these constructs were adapted from previously validated instruments reported in the literature on cognitive interference, environmental noise, attentional regulation, task engagement, and flow experience. Item wording was adjusted to fit the context of everyday learning activities, and the questionnaire was reviewed to ensure clarity and contextual appropriateness before the formal survey was conducted. For example, cognitive interference items asked respondents to indicate the extent to which they experienced distractions or competing thoughts during learning tasks (e.g., “I found my attention being drawn away from the task by other thoughts or interruptions”), while focused attention items assessed the ability to sustain concentration despite potential disturbances (e.g., “I was able to maintain my focus on the task even when distractions occurred”).

### 3.2 Participants and sampling

Data were collected from individuals engaged in everyday learning activities. Participants were recruited through convenience sampling from universities located in southern China in order to capture naturally occurring perceptions of distraction, attentional regulation, and immersive task experience during routine academic study. A total of 820 questionnaires were initially collected. After data screening, 173 responses were excluded due to incomplete questionnaires, substantial missing data, or unusually short completion times, resulting in 647 valid responses for the final analysis (valid response rate = 78.9%). The final sample consisted of 323 females (49.9%) and 324 males (50.1%). Participants were university learners engaged in typical study activities such as reviewing course materials, completing assignments, and preparing for examinations in natural learning environments (e.g., libraries, dormitories, and other self-directed study spaces). This context allowed the study to examine how perceptions of cognitive interference, attentional regulation, and flow experience emerge during everyday academic tasks.

### 3.3 Measures

All variables were measured using multi-item Likert-type scales adapted from established instruments in the literature and adjusted to the learning context of this study. Respondents were asked to evaluate their experiences during recent learning tasks. Cognitive interference was measured using items assessing perceived distraction, interruptions, and competing cognitive demands during task performance. Environmental noise was measured through items capturing the presence of background sound and sensory disturbances in the learning environment.

Focused attention was measured using items assessing attentional stability and the ability to maintain concentration despite potential distractions. Task engagement captured behavioral persistence, effort investment, and the tendency to resume task activity after interruptions. Flow experience was measured using items reflecting immersion, intrinsic involvement in the task, and altered perception of time during task activity. All items were rated on a five-point Likert scale ranging from 1 (strongly disagree) to 5 (strongly agree). The measurement reliability and convergent validity of the scales were assessed using Cronbach’s alpha, composite reliability (CR), and average variance extracted (AVE).

### 3.4 Data collection procedure

Data were collected using a structured questionnaire administered in natural learning settings. Participants were asked to reflect on specific recent learning tasks rather than general study habits, which helped reduce abstraction in responses and capture context-specific perceptions of distraction and engagement. The survey was administered anonymously, and respondents completed the questionnaire voluntarily.

### 3.5 Data analysis

All analyses were conducted using Python (version 3.11). Data processing and statistical analyses were performed programmatically to ensure reproducibility. Data cleaning and variable construction were implemented using standard data-processing libraries. Hierarchical regression models were estimated using ordinary least squares (OLS) routines from the statsmodels package. Heteroskedasticity-robust standard errors were calculated using the HC3 estimator. Indirect effects were tested using bootstrap resampling procedures implemented in Python. Measurement quality was assessed using reliability and validity indicators, including Cronbach’s alpha, composite reliability (CR), and average variance extracted (AVE).

### 3.6 Ethical considerations

Participation in the study was voluntary and anonymous. Respondents were informed about the purpose of the research before completing the questionnaire, and all responses were used solely for academic research purposes. No personally identifiable information was collected.

## 4 Results

### 4.1 Distributional profile of key variables

Table 1 reports descriptive statistics for the main study variables. The means of cognitive interference (X1), environmental noise (X2), focused attention (M1), task engagement (M2), and flow experience (Y) are all close to the midpoint of the measurement scale, with standard deviations around 0.70. This distribution suggests that participants generally experienced a moderate level of task disruption and maintained a moderate level of attentional focus and engagement during task performance. The observed ranges extend across most of the scale, indicating that while some participants reported relatively low or high levels on particular constructs, the majority of responses cluster around the central values. Overall, the data reflect a realistic task context in which individuals operate under noticeable but manageable levels of cognitive and environmental disturbance. One notable feature of the data is that both cognitive interference and focused attention remain at moderate levels on average. If interference merely reflected uncontrollable external noise, higher interference scores would be expected to correspond to sharply reduced attention. In the present sample, however, attention does not collapse when interference increases. Instead, the pattern suggests that individuals actively regulate their attention in response to disruptive conditions. Attention therefore appears less as a fixed cognitive capacity and more as a regulatory process that is repeatedly mobilized during task performance to maintain task focus. Task engagement also lies near the midpoint of the scale, suggesting that participants generally remain involved in their tasks while adjusting their level of effort in response to interruptions. Rather than working in a perfectly uninterrupted manner, individuals appear to maintain progress by returning to the task after disruptions and reorganizing their behavior to continue task execution. This pattern is consistent with contemporary digital learning and work environments, where interruptions and competing demands are common features of everyday activity. Flow experience shows a similar mid-range distribution. Immersion is neither consistently absent nor constantly present but appears intermittently during task performance. This pattern indicates that flow emerges in episodes within environments that also contain competing cognitive demands. In interference-rich contexts such as those represented in this sample, immersive experience is unlikely to arise automatically. Instead, it is likely to develop through sustained attentional regulation and ongoing behavioral effort. Consequently, the deployment of attention and engagement mechanisms becomes a key factor in determining whether moments of immersive task experience occur.

**TABLE 1:** Summary statistics of study variables.

Variable	Mean	SD	Min	Max
X1 cognitive interference	2.984	0.721	1.167	4.833
M1 focused attention	3.010	0.705	1.200	5.000
Y flow experience	3.000	0.715	1.000	4.833
X2 environmental noise	2.994	0.667	1.200	5.000
M2 task engagement	3.000	0.666	1.250	4.750

### 4.2 Reliability and convergent validity of measurement scales

Table 2 presents the reliability and convergent validity statistics for the measurement scales used in the study. Overall, the indicators suggest that the constructs demonstrate acceptable measurement quality according to commonly used empirical standards. Cronbach’s α values range from 0.840 to 0.906, composite reliability (CR) values range from 0.899 to 0.931, and the average variance extracted (AVE) for all constructs exceeds 0.63. These results indicate that the items within each scale show a reasonably consistent pattern of responses and capture a substantial proportion of the variance associated with their underlying constructs. Cognitive interference and flow experience display relatively high internal consistency, suggesting that the items used to represent these constructs reflect closely related aspects of perceived disruption and immersive task experience. Focused attention and environmental noise also demonstrate stable reliability levels, indicating that participants tend to report these situational conditions in a relatively coherent manner while still allowing for variation across different task contexts. Task engagement shows slightly lower, but still satisfactory, reliability compared with the other constructs. This pattern may reflect the nature of engagement as a behavioral regulation process that unfolds across task episodes and may not always manifest simultaneously in every situation. Taken together, the reliability and convergent validity indicators suggest that the measurement scales are sufficiently consistent for the purposes of the present analysis. While measurement limitations cannot be entirely ruled out, the observed values provide reasonable support for examining the relationships among cognitive interference, attentional regulation, task engagement, and flow experience in the subsequent analyses.

**TABLE 2:** Scale reliability and convergent validity.

Construct	Items	Cronbach’s α	CR	AVE
X1 cognitive interference	7	0.906	0.931	0.660
X2 environmental noise	6	0.868	0.911	0.633
M1 focused attention	6	0.889	0.922	0.667
M2 task engagement	5	0.840	0.899	0.644
Y flow experience	7	0.904	0.930	0.656

### 4.3 Zero-order associations among predictors, mechanisms, and flow experience

Table 3 presents the zero-order correlations among the focal constructs. Overall, the pattern of correlations suggests that the variables are systematically related while remaining empirically distinguishable. Cognitive interference shows a moderate positive correlation with focused attention (*r* = 0.584, *p* < 0.001), indicating that higher levels of perceived disruption tend to co-occur with higher levels of reported attentional focus. Rather than implying that interference directly enhances attention, this association may reflect a context in which individuals attempt to regulate their attention when disruptions arise. In such situations, attention may function less as a stable cognitive capacity and more as a regulatory process that is repeatedly mobilized during task performance. Cognitive interference is also positively associated with flow experience (*r* = 0.539, *p* < 0.001). Although flow is often described as emerging in relatively undisturbed environments, this pattern suggests that immersive experiences can still occur in settings where interference is present. In these contexts, flow may represent moments in which individuals successfully organize their attention and activity despite ongoing distractions. Focused attention shows the strongest correlation with flow experience among the predictors (*r* = 0.592, *p* < 0.001), which is consistent with the idea that immersion is closely related to the ability to maintain attentional stability during task execution. Environmental noise is moderately correlated with both attention (*r* = 0.500, *p* < 0.001) and flow experience (*r* = 0.520, *p* < 0.001), suggesting that external disturbance is part of the situational context within which attentional regulation and immersion unfold. In contrast, task engagement displays weaker correlations with the other constructs and shows almost no concurrent association with flow (*r* = 0.030). This pattern may indicate that engagement reflects a behavioral tendency to persist with task activity rather than an experiential state of immersion itself. Taken together, the correlations appear broadly consistent with a functional pattern in which interference and environmental conditions shape the context of task performance, attentional regulation plays a central role in maintaining focus, and moments of flow emerge when attention is successfully sustained despite ongoing disruption. These associations provide an initial empirical basis for the subsequent analyses examining how interference relates to flow through attention and task engagement.

**TABLE 3:** Correlations among focal constructs.

Variable	X1	M1	Y	X2	M2
X1 cognitive interference	1				
M1 focused attention	0.584***	1			
Y flow experience	0.539***	0.592***	1		
X2 environmental noise	0.450***	0.500***	0.520***	1	
M2 task engagement	−0.226***	−0.108**	0.030	0.019	1

The symbols indicate the levels of statistical significance: *p < 0.05, *p < 0.01, and **p < 0.001.

### 4.4 Incremental prediction of flow experience: evidence from hierarchical regression models

**FIGURE 1:**
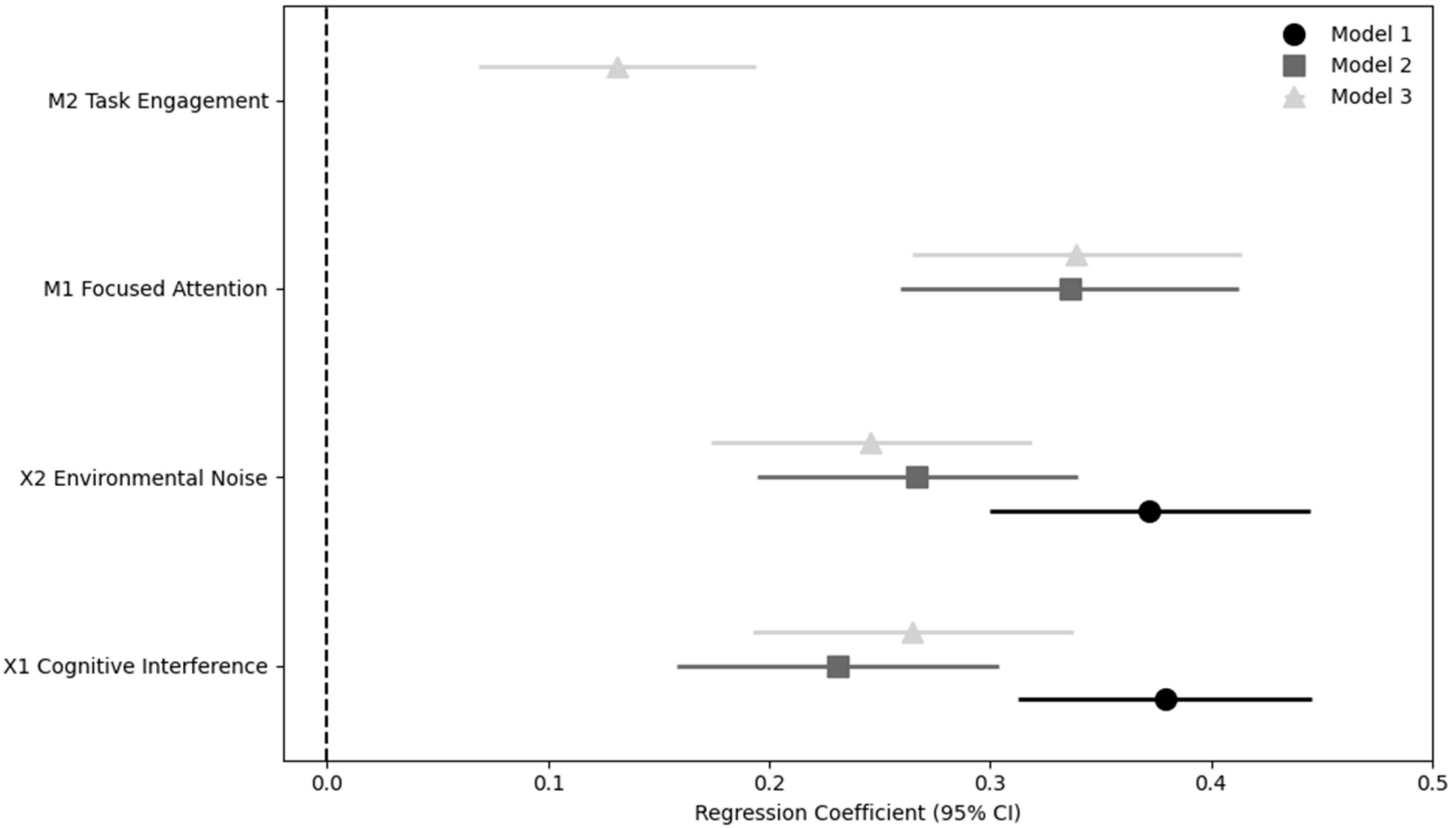
Coefficient plot of hierarchical regression models predicting flow experience.

Figure 1 and Table 4 present the hierarchical regression results predicting flow experience. In Model 1, cognitive interference and environmental noise are entered as predictors and together explain a moderate proportion of the variance in flow experience (*R*^2^ = 0.387). Both variables show positive and statistically significant coefficients, suggesting that higher levels of perceived disruption and environmental disturbance tend to be associated with higher reported levels of flow experience. This pattern indicates that immersive task experiences in the present sample do not occur only under conditions of minimal disturbance but may also arise in contexts where interruptions or competing stimuli are present. When focused attention is introduced in Model 2, the explanatory power of the model increases to *R*^2^ = 0.451, indicating that attentional regulation accounts for additional variance in flow experience beyond the influence of interference and environmental noise. At the same time, the coefficients for cognitive interference and environmental noise become somewhat smaller while remaining statistically significant, whereas focused attention shows a relatively strong positive association with flow. This pattern suggests that the relationship between interference-related conditions and immersive experience may partly depend on the way individuals regulate and maintain their attention during task performance. In Model 3, task engagement is added to the model, producing a further but relatively modest increase in explained variance (*R*^2^ = 0.465). Task engagement shows a positive and statistically significant coefficient, although its magnitude is smaller than that of focused attention, indicating that engagement may contribute to sustaining task activity but may play a comparatively smaller role in predicting the experiential aspect of immersion. Importantly, both cognitive interference and environmental noise remain significant predictors in the full model, suggesting that these contextual factors continue to relate to flow experience even after accounting for attentional regulation and behavioral persistence. Overall, the coefficient patterns illustrated in Figure 1 remain relatively stable across the hierarchical models: focused attention displays the strongest association with flow, task engagement shows a smaller but reliable relationship, and interference-related variables retain positive links with the outcome variable. Taken together, these results suggest that immersive task experiences in the present context are closely connected to attentional regulation and ongoing task involvement while emerging within environments in which interruptions and environmental disturbances remain present.

**TABLE 4:** Hierarchical regression models predicting flow experience.

Predictor	Model 1	Model 2	Model 3
X1 cognitive interference	0.379*** (0.034)	0.231*** (0.037)	0.265*** (0.037)
X2 Environmental noise	0.372*** (0.037)	0.267*** (0.037)	0.246*** (0.037)
M1 focused attention	–	0.336*** (0.039)	0.339*** (0.038)
M2 task engagement	–	–	0.131*** (0.032)
*R* ^2^	0.387	0.451	0.465
N	647	647	647

The symbols indicate the levels of statistical significance: *p < 0.05, *p < 0.01, and **p < 0.001.

### 4.5 Mechanism testing: bootstrap estimates of indirect effects

**FIGURE 2:**
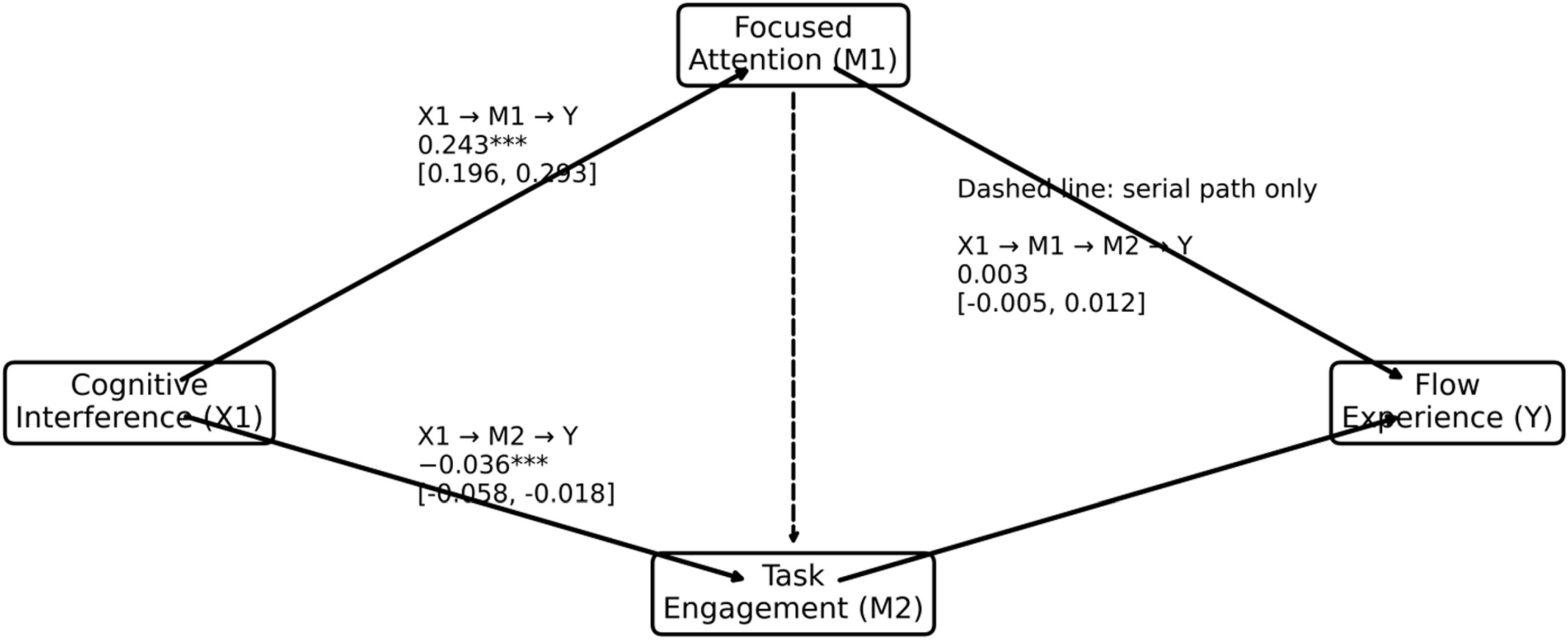
Mediation structure and bootstrap indirect effects. The symbols indicate the levels of statistical significance: *p < 0.05, *p < 0.01, and **p < 0.001.

Figure 2 and Table 5 report the bootstrap estimates of the indirect effects linking cognitive interference to flow experience through focused attention and task engagement. The results suggest that the largest indirect pathway operates through focused attention. The estimated indirect effect of cognitive interference on flow experience via focused attention is 0.243, and the 95% confidence interval does not include zero (0.196, 0.293), indicating that this pathway is statistically significant. This pattern suggests that higher levels of perceived interference may be associated with greater immersion partly through the way individuals regulate and stabilize their attention during task execution. In environments where interruptions and competing demands are present, maintaining attentional focus may create conditions under which moments of immersive task experience can emerge. The indirect pathway through task engagement alone shows a small negative effect (−0.036), with a confidence interval that also excludes zero (−0.058, −0.018). This result provides support for H4, although the indirect effect is small and negative. In contrast, the sequential mediation pathway through focused attention followed by task engagement yields a very small indirect effect (0.003), and its confidence interval includes zero (−0.005, 0.012), suggesting that this serial pathway is not statistically supported in the present data. These results therefore do not support H5. Taken together, the mediation results indicate that attentional regulation appears to play a central role in linking interference-related conditions to immersive task experience, whereas engagement may primarily reflect the continuation of task activity rather than serving as a direct psychological carrier of flow. Overall, the empirical findings support H1, H2, and H3, while H4 receives partial support due to the small and negative indirect effect. The sequential mediation proposed in H5 is not supported.

**TABLE 5:** Indirect effects from cognitive interference to flow experience (bootstrap estimates).

Indirect path	Effect	SE	95% CI
X1 → M1 → Y	0.243	0.024	[0.196, 0.293]
X1 → M2 → Y	−0.036	0.010	[−0.058, −0.018]
X1 → M1 → M2 → Y	0.003	0.004	[−0.005, 0.012]

### 4.6 Robustness of main estimates across alternative model specifications

Table 6 presents the robustness checks conducted using several alternative model specifications. The results show that the estimated relationships remain largely stable across the different estimation approaches. Whether standardized coefficients are used or robust standard errors are applied, the coefficients for the key predictors remain very similar in magnitude and direction. Cognitive interference consistently shows a positive coefficient of approximately 0.26 across specifications, while environmental noise remains close to 0.24. Focused attention displays the largest coefficient in each specification, with values around 0.33, whereas task engagement shows a smaller but consistently positive association with flow experience, ranging from approximately 0.12 to 0.13. The explanatory power of the model also remains stable, with the baseline model accounting for 46.5% of the variance in flow experience. These results suggest that the main associations identified in the baseline regression are not particularly sensitive to scaling choices or the use of robust standard errors. Although minor variations in coefficient size appear across specifications, the ordering of predictors and the direction of their relationships with flow experience remain unchanged. In particular, focused attention consistently shows the strongest association with flow, while both cognitive interference and environmental noise retain positive relationships with the outcome variable. Task engagement continues to display a smaller but statistically significant effect across the alternative specifications. Taken together, these robustness checks suggest that the observed relationships among cognitive interference, attentional regulation, task engagement, and flow experience are relatively stable in the present dataset and are unlikely to be driven by a particular modeling choice.

**TABLE 6:** Robustness checks across alternative model specifications.

Predictor	Baseline	Standardized	Robust SE
X1 cognitive interference	0.265***	0.268***	0.265***
X2 environmental noise	0.246***	0.230***	0.246***
M1 focused attention	0.339***	0.334***	0.339***
M2 task engagement	0.131***	0.122***	0.131***

The symbols indicate the levels of statistical significance: *p < 0.05, *p < 0.01, and **p < 0.001.

## 5 Discussion

### 5.1 Flow under persistent interference: revisiting classical flow assumptions

The findings related to H1 and H2 indicate that both cognitive interference and environmental noise remain significant predictors of flow experience in the present study. In the hierarchical regression models, these two variables retain positive coefficients even after focused attention and task engagement are introduced, while the model explains a substantial proportion of the variance in flow. These results suggest that immersive experience in the learning context examined here does not occur only in environments where disturbance is absent but may also emerge under conditions in which interference remains present. At first glance, this pattern appears to contrast with a large body of research showing that environmental noise and distraction disrupt cognitive processing and reduce task performance. Studies of auditory distraction demonstrate that background noise competes for attentional resources and weakens the stability of sustained cognitive activity (Beaman, 2005), while reviews of learning environments report that environmental noise can impair concentration and learning efficiency, particularly during cognitively demanding tasks (Klatte et al., 2013). From this perspective, interference is typically treated as a contextual condition that undermines the attentional stability required for effective task performance. However, research on workflow interruptions suggests that individuals rarely disengage completely from tasks when disruption occurs. Instead, they frequently reorganize their attention in order to resume ongoing task activity after interruptions (Baethge and Rigotti, 2013). In digitally mediated learning environments, learners are routinely exposed to multiple competing sources of information and distraction, which requires them to repeatedly redirect their attention during task execution (Rosen et al., 2011). Under such conditions, task performance may depend less on the elimination of interference and more on the ability to repeatedly restore attentional focus while interruptions remain present. From this perspective, the positive associations observed in the present study may reflect the dynamic regulatory processes through which individuals manage interference during task activity. Immersive experience may arise during periods in which attentional focus is temporarily stabilized despite the continued presence of distraction. This interpretation is consistent with findings showing that reducing distraction strengthens the relationship between flow experience and learning performance in virtual learning environments (Bian et al., 2022). Rather than representing a stable psychological state that emerges only in distraction-free environments, flow in contemporary learning settings may therefore occur in episodic form, appearing during intervals in which individuals successfully regain attentional stability between interruptions. Taken together, these findings support H1 and H2 and suggest that in interference-rich environments, flow may function as a dynamic experiential state that emerges when individuals temporarily stabilize their attention within cognitively demanding task contexts.

### 5.2 Attentional regulation as the central mechanism linking interference and flow

The results related to H3 indicate that focused attention plays a central role in linking interference conditions to flow experience. In the regression models, focused attention shows the strongest positive coefficient among all predictors, suggesting that attentional stability is closely associated with the emergence of immersion during task performance. The mediation analysis further indicates that cognitive interference is positively associated with flow experience through focused attention, suggesting that individuals may respond to disruptive conditions by actively regulating their attentional focus. In such contexts, higher perceived interference may reflect situations in which individuals are required to mobilize regulatory attention more actively in order to stabilize task processing. Rather than simply eliminating immersion, interference may therefore trigger additional attentional effort aimed at maintaining task continuity. Research in cognitive psychology has long demonstrated that attention can suppress the influence of distracting stimuli by selectively enhancing task-relevant information (Hopf et al., 2002). Studies on cognitive load further suggest that individuals can temporarily shield task processing from external distraction by allocating additional attentional resources to the focal activity (Sörqvist et al., 2016). From this perspective, the positive mediation effect observed in this study may reflect the role of attentional control in stabilizing task focus despite environmental disturbance. Research on auditory and environmental distraction similarly indicates that individuals often rely on cognitive control mechanisms to regulate the impact of unexpected stimuli (Parmentier and Hebrero, 2013), while neurocognitive studies show that attentional systems contribute to resolving interference created by competing emotional or environmental signals (Dolcos and McCarthy, 2006). In learning environments characterized by multiple competing stimuli, attentional regulation may therefore function as a key mechanism through which individuals maintain a coherent task experience. When learners repeatedly redirect their attention back to the task after interruptions, attentional stability may temporarily recover, allowing brief periods of immersion to emerge even in the presence of ongoing interference. Taken together, these findings support H3 and suggest that attentional maintenance represents a central regulatory process through which individuals can sustain flow experience in cognitively demanding learning environments.

### 5.3 Distinct roles of attentional maintenance and task engagement

The results related to H4 and H5 indicate that task engagement and focused attention play different roles in linking cognitive interference to flow experience. Although task engagement constitutes a statistically significant indirect pathway, this pathway is relatively small and negative, whereas the sequential pathway through focused attention and task engagement is not supported. The mediation analysis shows that task engagement contributes to the relationship between cognitive interference and flow experience, providing partial support for H4, although the magnitude of this pathway is modest compared with the stronger effect observed for focused attention. In contrast, the sequential mediation pathway linking cognitive interference, focused attention, task engagement, and flow experience is not statistically supported in the present data, suggesting that H5 is not confirmed.

This pattern suggests that attentional regulation and behavioral engagement represent related but functionally distinct processes in disruption-rich learning environments. While focused attention appears to be closely associated with the experiential dimension of immersion, task engagement may primarily reflect the behavioral continuation of task activity. In learning contexts characterized by frequent interruptions, individuals may continue working on tasks even when their subjective sense of immersion fluctuates. Research on student behavior in digitally mediated learning environments shows that learners often develop strategies to cope with distraction while maintaining task participation (Aagaard, 2021). Such strategies help sustain observable task involvement but do not necessarily guarantee stable attentional immersion. Similarly, studies of learning processes suggest that engagement in educational contexts may involve multiple behavioral and cognitive components that do not always correspond to a unified experiential state (Parker and Heywood, 2013). From this perspective, engagement may represent persistence in task activity rather than the psychological absorption typically associated with flow. Research on distraction further indicates that individuals sometimes rely on behavioral continuation as a way to manage disruptive stimuli without fully eliminating their cognitive effects (Senn and Radomsky, 2015). As interference increases, individuals may therefore remain behaviorally engaged in the task while experiencing fluctuations in attentional stability and immersive experience. This dynamic may explain why the indirect pathway through task engagement is comparatively weaker and negative in the present model, whereas focused attention shows a stronger and more consistent association with flow. Taken together, these findings suggest that attentional maintenance and task engagement represent two complementary but distinct regulatory processes through which individuals manage interference during task performance.

### 5.4 Practical implications, limitations, and directions for future research

The findings of this study also carry several practical implications for learning and work environments characterized by frequent interruption and competing stimuli. In many contemporary digital learning contexts, individuals are required to manage multiple sources of information simultaneously while maintaining task focus. Research on distraction in technology-mediated learning suggests that learners often need to actively regulate their attention in order to sustain productive task engagement in such environments (Aagaard, 2021). The present results suggest that improving learning experiences may therefore depend not only on reducing external disturbance but also on supporting learners’ ability to stabilize their attention during task performance. Educational design strategies that reduce unnecessary distractions, structure tasks more clearly, and support attentional regulation may help increase the likelihood of immersive learning experiences. At the same time, the findings indicate that behavioral engagement alone may not guarantee the emergence of flow. Learners may continue working on tasks while their attentional stability fluctuates, suggesting that effective learning environments should support both sustained participation and attentional coherence.

Several limitations should also be acknowledged when interpreting the results of this study. First, the data are based on self-reported measures collected in a specific learning context, which may limit the generalizability of the findings to other environments or populations. Self-report measures are commonly used in studies of task experience and attention, but they may not fully capture the dynamic nature of cognitive processes during task performance. Second, the cross-sectional design of the study limits the ability to draw strong causal conclusions about the relationships among interference, attentional regulation, and flow experience. Future studies may benefit from longitudinal or experimental designs that track how attentional regulation and immersion evolve over time in response to changing task conditions. Third, the present study focuses primarily on cognitive and environmental interference during learning tasks, whereas other factors such as emotional states, motivational processes, or collaborative dynamics may also influence the emergence of immersive experience. In addition, although the findings support H1 and H2 by showing positive associations between interference-related conditions and flow experience, these results should be interpreted with caution. The present study does not suggest that cognitive interference or environmental noise are inherently beneficial for learning or task performance. Rather, the results may reflect the regulatory processes through which individuals manage disruption in environments where distraction is already a routine feature of task activity. In such contexts, higher perceived interference may coincide with greater mobilization of attentional resources, allowing immersion to emerge episodically when attentional stability is temporarily restored. Future research may therefore further investigate how different forms of distraction interact with attentional control and task engagement across various learning contexts. Experimental studies that manipulate environmental noise or task interruption could provide stronger evidence regarding the mechanisms through which attention supports immersion under interference. Advances in neurocognitive and behavioral measurement may also allow researchers to capture the moment-to-moment dynamics of attention and distraction more precisely. In addition, future work may explore how instructional design, digital learning tools, or workspace environments can be optimized to reduce unnecessary distraction while supporting learners’ ability to re-establish focus after interruption.

Despite these limitations, the present study contributes to a growing body of research that examines how individuals sustain meaningful task experience in environments characterized by cognitive and environmental complexity. By demonstrating that interference and immersion can coexist within the same task context, the findings suggest that flow in contemporary learning environments may often emerge as a dynamic regulatory experience rather than as a stable state achieved only in distraction-free settings. Understanding how attentional regulation enables individuals to maintain coherent task experience under persistent interference may therefore represent an important step toward designing learning and work environments that support both cognitive performance and meaningful engagement. This perspective extends existing research by showing that flow can emerge as a dynamic regulatory experience rather than as a state dependent on distraction-free environments.

## 6 Conclusion

This study examined how flow experience can be sustained in learning environments characterized by persistent cognitive interference and environmental noise. Drawing on survey data from 647 participants, the findings show that both cognitive interference and environmental noise remain significant predictors of flow experience even after attentional and behavioral regulatory mechanisms are incorporated into the model. These results suggest that immersive task experience in contemporary learning environments does not necessarily depend on the complete absence of distraction. Instead, flow may emerge when individuals actively regulate their attention in the presence of competing stimuli. The analysis further indicates that focused attention plays a central mediating role linking interference to flow experience, highlighting the importance of attentional regulation in stabilizing task focus under disruptive conditions. When interruptions occur, individuals may repeatedly redirect their attention back to the task, allowing short periods of immersion to arise despite ongoing cognitive demands. In contrast, task engagement demonstrates a weaker and less consistent relationship with flow, suggesting that behavioral persistence and experiential immersion represent related but distinct aspects of task performance. Learners may therefore continue participating in tasks even when attentional stability fluctuates, resulting in situations where task activity persists while the intensity of immersive experience varies. Overall, these findings contribute to a more nuanced understanding of flow in contemporary learning environments. Rather than representing a stable psychological state that appears only in distraction-free settings, flow may be better understood as a dynamic regulatory experience that emerges through repeated cycles of distraction, attentional recovery, and renewed task focus. By clarifying the distinct roles of attentional regulation and behavioral engagement under persistent interference, this study extends existing research on attention, distraction, and learning, and provides new insight into how immersive experience can be sustained in cognitively demanding environments. These findings also suggest that future research and educational design may benefit from focusing not only on reducing external distractions but also on supporting learners’ capacity to restore attentional stability and re-engage with tasks in complex digital learning contexts.

## Data Availability

The raw data supporting the conclusions of this article will be made available by the authors, without undue reservation.
